# Mechanisms, Hallmarks, and Implications of Stem Cell Quiescence

**DOI:** 10.1016/j.stemcr.2019.05.012

**Published:** 2019-06-11

**Authors:** Inchul J. Cho, Prudence PokWai Lui, Jana Obajdin, Federica Riccio, Wladislaw Stroukov, Thea Louise Willis, Francesca Spagnoli, Fiona M. Watt

**Affiliations:** 1 Centre for Stem Cells and Regenerative Medicine, King's College London, Guy's Hospital, Floor 28, Tower Wing, Great Maze Pond, London SE1 9RT, UK

**Keywords:** stem cells, cancer, quiescence, cell cycle, autophagy, niche, cell therapy

## Abstract

Cellular quiescence is a dormant but reversible cellular state in which cell-cycle entry and proliferation are prevented. Recent studies both *in vivo* and *in vitro* demonstrate that quiescence is actively maintained through synergistic interactions between intrinsic and extrinsic signals. Subtypes of adult mammalian stem cells can be maintained in this poised, quiescent state, and subsequently reactivated upon tissue injury to restore homeostasis. However, quiescence can become deregulated in pathological settings. In this review, we discuss the recent advances uncovering intracellular signaling pathways, transcriptional changes, and extracellular cues within the stem cell niche that control induction and exit from quiescence in tissue stem cells. We discuss the implications of quiescence as well as the pharmacological and genetic approaches that are being explored to either induce or prevent quiescence as a therapeutic strategy.

## Teaser

Stem cell quiescence is a dormant but reversible cellular state in which cell-cycle entry and proliferation are prevented. Intrinsic regulators include the cell-cycle machinery, autophagy, chromatin modifiers, and microRNAs. Extrinsic regulators include cell-adhesion molecules and secreted factors. Therapeutic modulation of stem cell quiescence could restore tissue homeostasis.

## Main Text

### Introduction

Quiescent cells are non-dividing and exist in the G_0_ stage of the cell cycle in a temporary and reversible manner (Daignan-Fornier and Sagot, 2011). This state is distinct from senescence, whereby cells are irreversibly arrested in G_0_, ultimately leading to degeneration and cell death (Terzi et al., 2016). Studies in yeast, where quiescence serves as a survival response to adverse environmental conditions, have provided insights into how different signals integrate to regulate entry into the quiescent state (Brauer et al., 2008, Dhawan and Laxman, 2015). In adult mammalian tissue stem cells (SCs), quiescence can be essential for survival and long-term tissue maintenance and regeneration. Initially it was believed—based largely on classic studies of hematopoietic SCs (HSCs)—that quiescence was an integral property of all SCs, allowing them to preserve their proliferative potential and limit DNA damage. However, the discovery of highly proliferative SCs in many tissues has challenged this notion (Clevers and Watt, 2018).

Tight control over SC quiescence entry and exit is actively maintained through intrinsic mechanisms, systemic factors, and interactions with the microenvironment. Cells in this state are characterized by lower metabolic activity, generating ATP via the glycolytic pathway rather than oxidative phosphorylation (Arai and Suda, 2008), as well as suppressed transcription and translation (Oulhen et al., 2017). Gene expression profiles across different populations of quiescent SCs, such as HSCs (Forsberg et al., 2010), muscle SCs (MuSCs) (Fukada et al., 2007), and hair follicle SCs (HFSCs) (Blanpain et al., 2004), show a downregulation of genes involved in DNA replication, cell-cycle progression, proliferation, and mitochondrial function. In contrast, an upregulation of genes involved in cell-fate decisions is observed (Cheung and Rando, 2013).

Reversibility of quiescence allows rapid reactivation of the cell cycle, achieved through mechanical cues, soluble factors, and intracellular signaling pathways (Quarta et al., 2016). The activation of quiescent SCs can lead to generation of transit-amplifying progenitors (TAPs) that in turn give rise to committed progeny, which regenerate damaged tissue or participate in tissue turnover (Evano and Tajbakhsh, 2018). Inevitably, deregulation of quiescence can lead to aberrant differentiation, apoptosis, or senescence, disrupting tissue homeostasis and impairing tissue regeneration (Evano and Tajbakhsh, 2018).

This review examines the extrinsic and intrinsic mechanisms governing the maintenance of SC quiescence and entry and exit from the cell cycle, as well as the implications for disease. Finally, we evaluate pharmacological and genetic approaches to modulating quiescence as a therapeutic strategy. We focus on MuSCs, HSCs, HFSCs, and neural SCs (NSCs), because the role of quiescence is well established in these cell types.

### Intrinsic Factors Regulating Stem Cell Quiescence

Quiescence entry/exit and maintenance are orchestrated by a combination of processes including transcriptional regulation of cell-cycle genes, chromatin modification, and microRNA (miRNA)-mediated control of gene expression. Entry into quiescence tends to be associated with an alteration in metabolic activity regulated by mitochondrial biogenesis genes and macroautophagy.

#### Cell-Cycle Regulators

The core intrinsic mechanisms governing cellular quiescence primarily restrict cell-cycle progression. RB (retinoblastoma protein) is one of the main suppressors of E2F transcription factor activity, making it a central player in mediating G_1_/S transition (Trimarchi and Lees, 2002). RB-E2F signaling is highly conserved, and in *Drosophila* testis knockdown of RBF (pRB homolog) results in active proliferation of quiescent hub cells, the signaling center for germline stem cell recruitment. On double knockdown of RBF and dE2F1 there is no active hub cell proliferation, and normal population size is restored (Greenspan and Matunis, 2018). RB-E2F signaling also plays an essential role in mammalian stem cell maintenance. Knockout of all RB proteins drives hyperproliferation in HSCs and early hematopoietic progenitors (Viatour et al., 2008). Despite not affecting HSC short-term self-renewal ability, these deletions impair HSC long-term capability to restore the hematopoietic system (Viatour et al., 2008). Ablation of RB *in vivo* also expands MuSC and myoblast populations, impairing their differentiation capacity (Hosoyama et al., 2011). In contrast, RB deletion increases proliferation of differentiated progenitors, such as olfactory neuroblasts (Jaafar et al., 2016) and hippocampal dentate gyrus granule cells (Vandenbosch et al., 2016), without affecting quiescent neural SCs.

**Figure 1:**
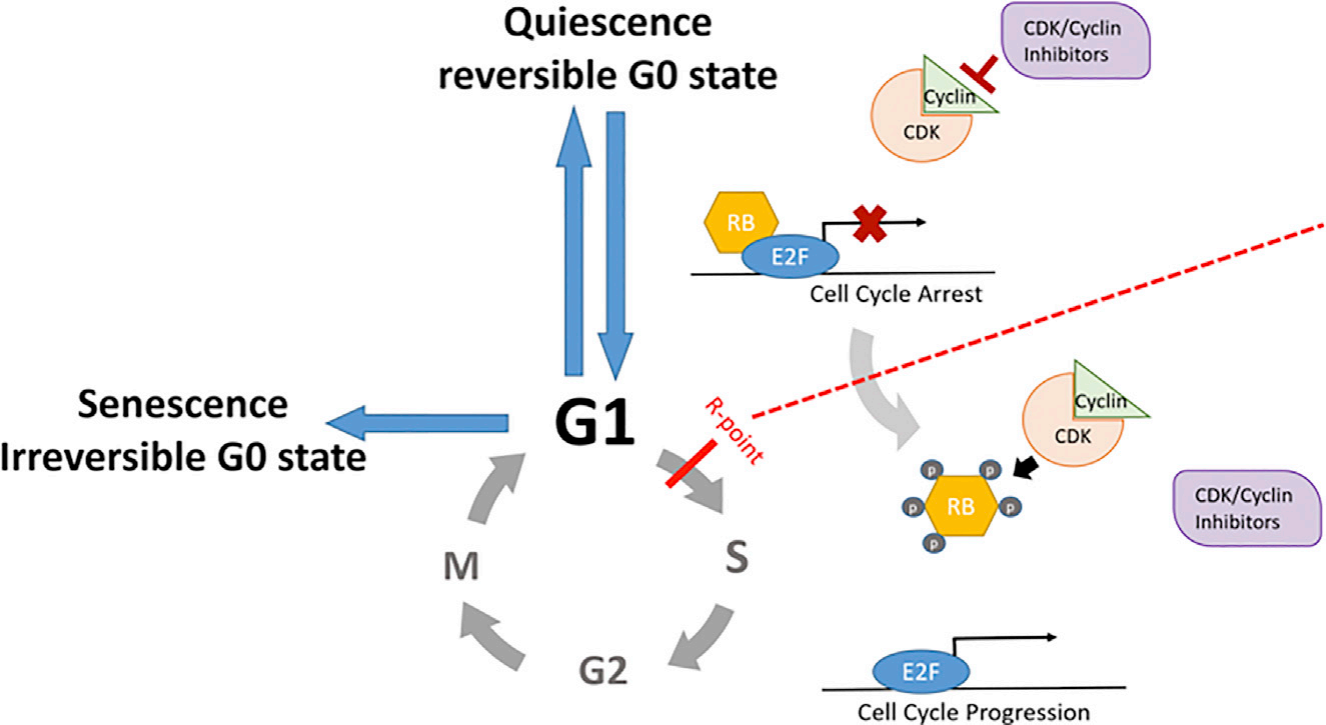
Quiescence (G_0_) Quiescence is a reversible G_0_ state, because cells retain the ability to re-enter G_1_ of the cell cycle after passing the restriction point (R-point) of the G_1_/S transition. Cells in G_1_ can also enter senescence, which is an irreversible state. E2F mediates transcription of cell-cycle genes. In quiescent cells, E2F is repressed by retinoblastoma (RB) binding. The repressive ability of RB is regulated by the CDK/cyclin complex, which in turn is controlled by CDK/cyclin inhibitors. Adapted from Biggar and Storey (2009).

RB is negatively regulated by heterodimeric complexes of cyclin proteins and CDK (cyclin-dependent kinases). Single knockouts of *Cdk2*, *Cdk4*, or *Cdk6* each affect tissue-specific proliferation in mice (reviewed in Malumbres and Barbacid, 2009). Differential expression of *Cdk6* underlies heterogeneity in the quiescence of human HSCs and modulates the frequency of HSC division (Laurenti et al., 2015). Knockdown of *CCNC* (Human Cyclin C gene) in HSCs increases the quiescent SC pool (Miyata et al., 2010). The involvement of CDK/cyclin complexes in mediating SC quiescence is also demonstrated by the effects of CDK inhibitors (*p21*^*cip1/waf1*^, p27^kip1^, and *p57*^*kip2*^), which regulate the switch between proliferation and quiescence (Spencer et al., 2013). Knockout of *p21*^*cip1/waf1*^ leads to increased proliferation and depletion of HFSCs (Lee et al., 2013) and HSCs (Berthet et al., 2007). Likewise, knockout of p27^Kip1^ results in a loss of quiescent radial glial SCs and an increase in neuroblasts re-entering the cell cycle (Ogawa et al., 2017). Conditional knockout of *p57*^*kip2*^ leads to a significant reduction in quiescent HSCs due to a decrease in phosphorylated RB (Matsumoto et al., 2011), subsequently increasing the amount of active E2F. Similarly, long-term depletion of *p57*^*kip2*^ leads to NSC exhaustion (Furutachi et al., 2013). Together, these studies highlight the importance of tight control over cell-cycle progression in regulating SC quiescence (Figure 1).

p53, a central player in apoptosis, senescence, and cell-cycle arrest (Kaiser and Attardi, 2018), is also involved in cellular quiescence. HSCs and NSCs from p53^−/−^ mice have a higher proliferation rate than those in control mice (Liu et al., 2009, Meletis et al., 2006). Conversely, overexpression of p53 arrests *ex vivo* MuSCs in a quiescent state (Flamini et al., 2018). p53 levels also regulate the differentiation potential and quiescence state of airway epithelial progenitors *in vivo* (McConnell et al., 2016), suggesting that p53 may function as a general regulator of SC quiescence.

#### Metabolic Regulation

A suppressed metabolic rate in quiescent cells is believed to retain nutrients and maintain low reactive oxygen species (ROS) production. To achieve this, the environmental sensing target of rapamycin pathway becomes inactive, leading to increased macroautophagy and a decrease in mitochondria (Valcourt et al., 2012).

**Figure 2:**
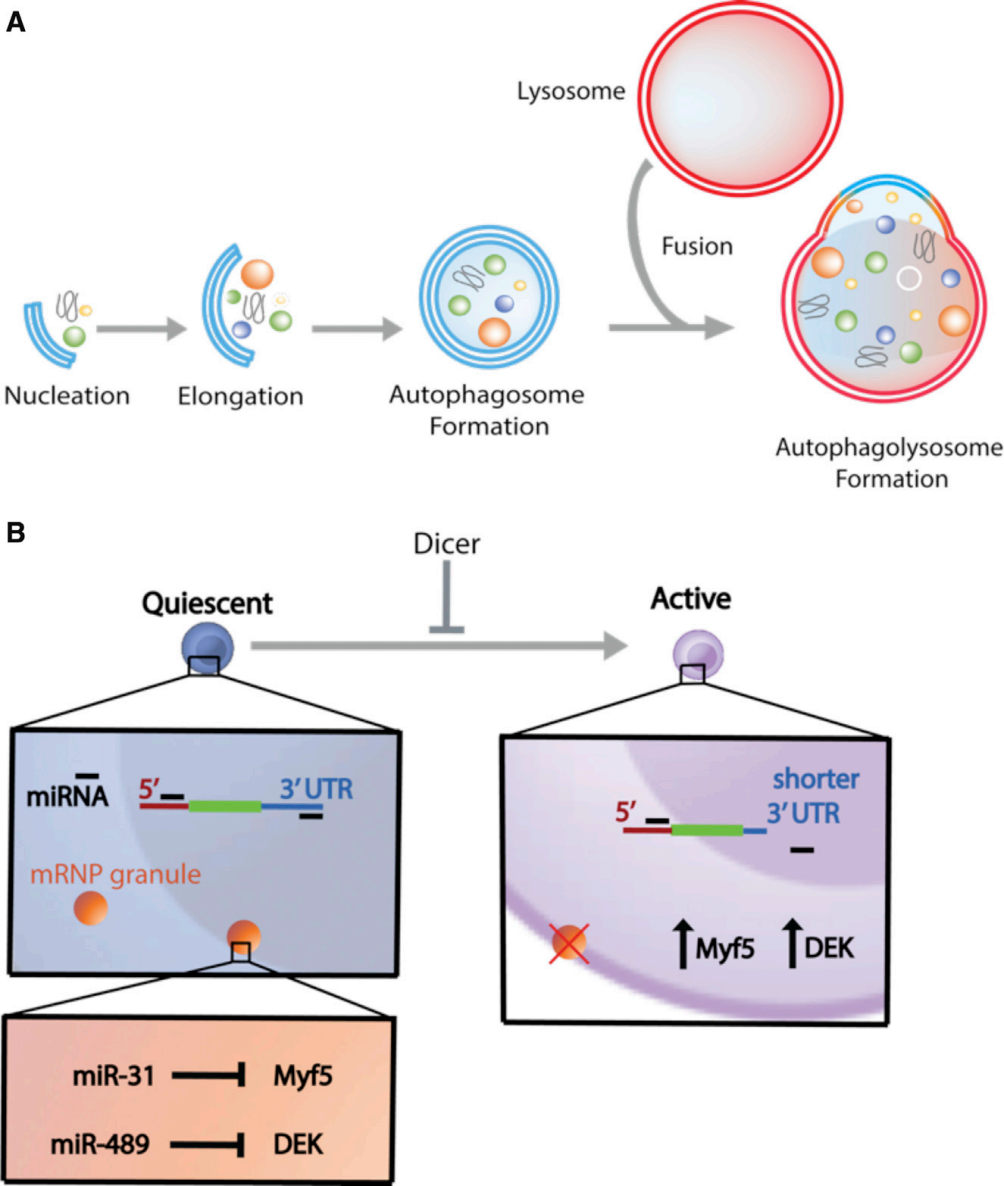
Intracellular Mechanisms Regulating Quiescence (A) Autophagy is an intracellular metabolic process characterized by the nucleation of a double-membrane vesicle termed the phagophore, which matures into the autophagosome. (B) Quiescence can be positively regulated by miRNA molecules that are produced by the endoribonuclease Dicer. miRNAs can bind the 3′ untranslated region (UTR) (blue) or 5′ seed region (red) of mRNA. Reduction in mRNA 3′ UTR length results in a release from miRNA inhibition. Sequestration of Myf5 and DEK, proteins promoting differentiation and proliferation respectively, occurs in quiescent muscle stem cells through messenger ribonucleoprotein (mRNP) granules that contain miRNAs such as miR-489 and miR-31.

Macroautophagy is a process of intracellular degradation characterized by the formation and elongation of a phagophore that engulfs cytoplasmic components to form an autophagosome. Fusion of the autophagosome with a lysosome allows for the recycling of cargo to sustain cell survival (Figure 2A). An increase in this recycling or “self-eating” process increases free nutrients and subsequently allows cells to decrease their metabolic rate, thereby maintaining quiescence (Ho et al., 2017). Additionally, through random engulfment, macroautophagy leads to elimination of ROS and toxic waste. Differing ROS levels are known to influence cell fate (Bigarella et al., 2014), with an increase in ROS resulting in a loss of quiescence and self-renewal in HSCs (Takubo et al., 2010). However, with age comes a decline in macroautophagy, resulting in a decrease in quiescent SC populations and an increase in senescence (Wen and Klionsky, 2016). Macroautophagy appears to act as a gatekeeper of quiescence in many SCs, including HSCs and MuSCs (Garcia-Prat et al., 2016), suggesting that restimulation of macroautophagy could rejuvenate aged quiescent SCs (Ho et al., 2017).

Direct suppression of metabolism is achieved through removal of mitochondria by repression of mitochondrial biogenesis/function genes (Chen et al., 2008, Li and Bhatia, 2011) via macroautophagy, or selective mitochondrial autophagy, also termed mitophagy. Mitochondrial biogenesis activates SCs and an aberrant increase in mitochondrial function results in the loss of quiescence (Gaucher et al., 2018). Furthermore, loss of pyruvate dehydrogenase kinases, which mediate non-canonical ATP production by anaerobic glycolysis, results in increased mitochondrial metabolism, attenuated HSC quiescence, and reduced transplantation capacity of HSCs (Takubo et al., 2013). Nevertheless, maintenance of some mitochondrial function in quiescent cells is essential, since inactivation of the RISP (Rieske iron-sulfur protein) subunit of mitochondrial complex III results in impaired respiration and subsequent loss of quiescence in adult HSCs (Ansó et al., 2017).

#### Epigenetics and miRNAs

There is strong evidence for differences in the epigenetic landscape of quiescent and active SCs. One such epigenetic mark is chromatin methylation, which is coordinated by repressive Polycomb group (PcG) and activating mixed-lineage leukemia (MLL) proteins. PcG proteins recruit PcG-repressive complexes (PRC1/2), triggering H3 lysine 27 trimethylation (H3K27me3) to suppress gene expression, whereas MLL proteins mediate H3K4me3 to antagonize gene repression (Srivastava et al., 2010). Besides H3K27me3, H3K9me3 and H4K20me2 are two histone methylation marks associated with gene repression (Kheir and Lund, 2010).

Loss of epigenetic modifiers, including H3K27me3 controlled by the p38/mitogen-activated protein kinase (MAPK) signaling pathway or H4K20 dimethyltransferase Suv4-20h1, increase expression of MuSC differentiation regulators, such as PAX7 and *MyoD*, leading to exit from quiescence and expansion of activated MuSCs (Boonsanay et al., 2016, Palacios et al., 2010). Similarly, a deficiency in PcG proteins, such as YY1, results in abolition of H3K27me3 modification and reduces quiescent HSCs (Lu et al., 2018). These observations suggest that the chromatin methylation landscape plays an important role in restricting differentiation and maintaining SCs in the quiescent state.

miRNAs also possess the ability to modulate gene expression, and several quiescent SC populations (NSCs, HSCs, MuSCs, and HFSCs) have a common miRNA profile, suggesting a role for miRNA regulation in quiescence (Arnold et al., 2011). Canonically, miRNA molecules bind a “seed region” on mRNA near the 5′ terminus. However, 3′ untranslated regions (UTRs) of gene transcripts are also miRNA targets. Reduction in 3′ UTR length, through mutations over time, or by alternate splicing and cleavage, can prevent miRNA inhibition of target genes, resulting in increased proliferation and decreased quiescence in many cell types (Figure 2) (Mayr and Bartel, 2009). Ablation of Dicer, the enzyme necessary for miRNA production, disrupts quiescence and causes premature apoptosis of MuSCs (Cheung et al., 2012). Furthermore, deletion of Dicer in the epidermis during the hair follicle growth phase (anagen) restrains the ability of HFSCs to enter the temporary rest phase (catagen) and subsequently results in loss of the SC pool (Teta et al., 2012). miR-489, a quiescence-specific miRNA, represses MuSC proliferation and increases quiescence through oncogene DEK suppression (Cheung et al., 2012). Similarly, Myf5 mRNA is inhibited by miR-31 through sequestration in messenger ribonucleoprotein (mRNP) granules in quiescent MuSCs. Removal of mRNP sequestration enables release of Myf5 mRNA, exit from quiescence, and subsequent myogenesis (Figure 2B). These observations support the hypothesis that quiescent SCs may exist in a poised state, primed for differentiation (Crist et al., 2012).

### Extrinsic Signals Regulating Quiescence of Adult Stem Cells

**Figure 3:**
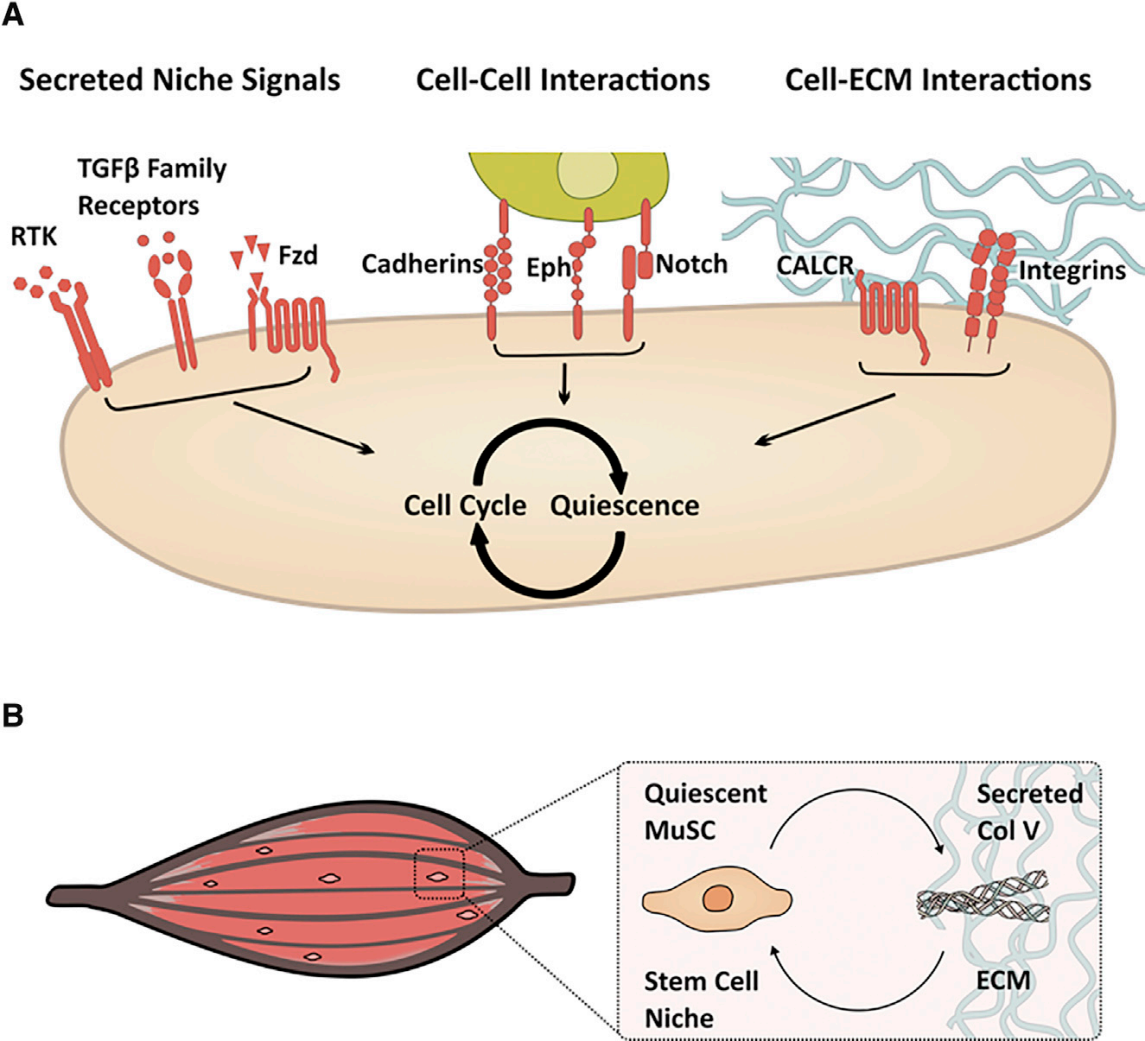
Microenvironmental Regulators of Quiescence (A) The stem cell microenvironment comprises multiple components, including direct interactions with neighboring cells, soluble factors, and binding sites on ECM proteins. Stem cells transduce those cues via their surface receptors and integrate the signals in complex intracellular regulatory networks. (B) In one example of microenvironmental modulation of quiescence, muscle stem cells secrete collagen V, which can act reciprocally via the calcitonin receptor (CALCR) to maintain quiescence.

Environmental cues such as nutrient deprivation or contact inhibition can induce reversible arrest of the cell cycle in yeast (Brauer et al., 2008, Dhawan and Laxman, 2015) or cultured mammalian cells (Coller et al., 2006). Quiescence mechanisms *in vivo* are likely to be more complex and dependent on the specialized microenvironment, described as the niche, in which SCs reside (Lane et al., 2014). Components of the SC microenvironment include other cell types that secrete soluble factors or modulate SC function via direct cell-cell contact. Adhesion to the extracellular matrix not only provides structural support but also regulates SC behavior (Figure 3A).

#### Direct Cell-Cell Interactions

A variety of cell-surface receptors mediate contact with other cell types and short-range intercellular communication via membrane-bound ligands, including Notch, Eph, and N-cadherin. The Notch pathway plays a key role in many aspects of tissue development and maintenance. For example, blocking Notch activity by conditional deletion of its downstream effector RBP-Jκ results in MuSC exit from quiescence and terminal differentiation (Philippos et al., 2012). Constitutive activation of the Notch pathway by overexpression of the intracellular domain of the Notch protein prevents SC depletion (Yue et al., 2017) and further blocks myogenic differentiation (Wen et al., 2012). Adhesive junctions between MuSC and muscle fibers mediated by homophilic interactions between cadherins also play a role in maintaining a quiescent state (Goel et al., 2017). Similarly, N-cadherin-mediated adhesion of NSCs and ependymal cells maintains NSC quiescence (Porlan et al., 2014).

In the subventricular zone (SVZ) of the brain, quiescence is induced by specialized protrusions in physical contact with endothelial cells of the perivascular niche. The endothelial and membrane-bound ligands Jagged1 (JAG1) and EphrinB2 activate Notch and Eph signaling, respectively, and act as negative regulators downstream of the MAPK pathway to attenuate proliferation signals from soluble growth factors (Ottone et al., 2014). External environmental cues can act via hippocampal dentate granule cells mediated by Eph signaling to switch between a proliferative and quiescent state of NSCs and thereby promote neurogenesis (Dong et al., 2019). Conversely, disruption of Eph signaling causes exit from quiescence and re-entry into the cell cycle (Conover et al., 2000).

In contrast to its quiescence-promoting function in NSCs and MuSCs, the interaction of the HFSC Notch receptor with tissue resident regulatory T cells induces proliferation of HFSC and regeneration of hair growth (Ali et al., 2017). This illustrates the context-dependent role of the Notch pathway in different tissues and cell types.

#### Secreted Factors

Quiescent SCs residing in their niches are exposed to a variety of secreted factors that regulate cell function, fate decisions, and quiescence. WNT signaling has been implicated in regulating tissue homeostasis, maintenance, self-renewal, and differentiation of the majority of SCs in adult tissues by three distinct pathways, canonical WNT/β-catenin activation, the non-canonical planar cell polarity pathway, and the WNT/Ca^2+^ pathway (Clevers, 2006). In NSCs, the balance between canonical and non-canonical WNT activity maintains SC identity and quiescence. Non-canonical signaling induces quiescence of NSCs in the SVZ via activation of the RhoGTPase CDC42, thereby promoting anchorage to the niche and upregulating expression of Notch1 and N-cadherin (Chavali et al., 2018). WNT-responding HFSCs in the outer bulge secrete autocrine WNT signals to maintain stem cell potency during the quiescent phase of the hair growth cycle and are marked by expression of the WNT target gene *Axin2*. Simultaneously, those cells secrete paracrine-acting WNT inhibitors such as Dickkopf (DKK) and Secreted frizzled-related protein 1 (SFRP1) to promote differentiation of cells in the inner hair follicle bulge, which results in a compartmentalization of the hair bulge layers (Lim et al., 2016).

Growth factors that act through receptor tyrosine kinases (RTKs) regulate the maintenance of SCs and can trigger quiescence exit to promote proliferation and differentiation. Fibroblast growth factors (FGFs) are known activators of MuSCs, and increased FGF signaling causes loss of quiescence and SC depletion (Chakkalakal et al., 2012). Release of hepatocyte growth factor after injury results in the transition of quiescent MuSCs to the primed “G_Alert_ state” and re-entry into the cell cycle (Rodgers et al., 2014). Conversely, HSCs in the BM niche that express the RTK TIE2 ligated with Angiopoietin-1 (ANG1) maintain their quiescent state and adhere to the HSC niche by upregulation of N-cadherin and integrins (Arai et al., 2004).

The transforming growth factor β (TGF-β) superfamily of growth factors, including bone morphogenetic proteins (BMPs), regulate key events in development and adult SC function (Watabe and Miyazono, 2009). BMPs are known regulators of neurogenesis in the dentate gyrus of the adult hippocampus (Colak et al., 2008). Non-dividing NSCs express the BMP receptor IA (BMPR-IA) and its effector SMAD4, which are subsequently downregulated in actively dividing progenitors. Inhibition of BMP signaling by its antagonist Noggin induces G_0_ cells to re-enter the cell cycle, thereby prematurely depleting the NSC pool (Mira et al., 2010). TGF-β1 signaling maintains NSC quiescence and simultaneously promotes survival of newly generated neurons while inhibiting progenitor proliferation by directly targeting cell-cycle regulators (Kandasamy et al., 2014). Thus, modulation of TGF-β signaling exerts distinct effects on different cell populations and limits NSC quiescence and differentiation to restricted regions within the niche.

#### Cell-Extracellular Matrix Interactions

Most cells in the body are in contact with extracellular matrix (ECM) proteins, which can be organized in a variety of ways, from basement membranes to collagenous fibers. The ECM provides attachment sites for residing SCs via specific receptors and ensures tissue integrity and cell survival (Gattazzo et al., 2014, Watt and Huck, 2013). Besides its role in anchoring cells within a tissue, the ECM plays an active role in regulating SC function. Secreted growth factors are sequestered by proteoglycans and other ECM proteins, thereby modulating their diffusion, activity, and availability to SCs (Schultz and Wysocki, 2009).

Disruption of tissue integrity through injury results in activation and release of stored growth factors (Miyazawa, 2010) capable of modulating the quiescent state of SCs (Rodgers et al., 2014). In addition, ECM proteins can exert a direct effect on quiescence. For instance, ECM deposition after wound healing stimulates the cellular switch in fibroblasts from a proliferating to a quiescent state (Rognoni et al., 2018). Moreover, the ECM protein Periostin promotes maintenance of quiescent HSCs via integrin signaling and downstream inhibition of the PI3K-AKT pathway (Khurana et al., 2016).

Integrins are central mediators of ECM signals to SCs and their regulation can impinge on additional pathways, creating complex regulatory networks and regulating the cell cycle (Assoian and Schwartz, 2001). β1-Integrins in MuSCs are involved in sensing SC niche signals and cooperating with the FGF pathway through mutual downstream effectors to regulate quiescence and activation, and deletion of the β1-integrin gene results in cell polarity defects and loss of quiescence (Rozo et al., 2016). MuSCs remodel the ECM niche by producing collagen V as a result of Notch signaling. Collagen V acts through the G-protein-coupled receptor (GPCR)/calcitonin receptor (CALCR) to retain MuSCs in the SC niche and delay cell cycling. Upon activation from quiescence, MuSCs reduce collagen V production. Inhibition of collagen V synthesis leads to activation and differentiation, illustrating that SCs can employ cell-autonomous mechanisms to maintain their own quiescence (Baghdadi et al., 2018) (Figure 3B).

In several tissues, including the small intestine and the hair follicle (Barker et al., 2010, Ito et al., 2005), quiescent SCs are compartmentalized separately from cycling SCs. This suggests the presence of distinct and specialized niches able to maintain the two different states (Latil et al., 2012). While in some cases compartmentalization is likely to be determined by ECM adhesion, there are additional determinants. For example, low oxygen levels induce HSC and NSC quiescence (Hermitte et al., 2005, Santilli et al., 2010), potentially via controlling ROS levels, and in the murine bone marrow, poorly vascularized hypoxic regions are populated by quiescent HSCs (Kubota et al., 2008, Sgarbi et al., 2018).

### Exploiting Quiescence for Novel Therapies

**Figure 4:**
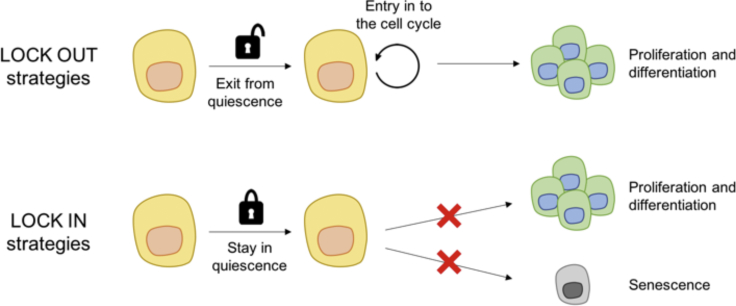
Modulation of Stem Cell Quiescence for Therapeutic Benefit “Lock-out” strategies consist of forcing stem cells out of quiescence to promote proliferation and differentiation. “Lock-in” strategies consist of re-establishing or maintaining the quiescent state to prevent aberrant proliferation and differentiation or premature senescence.

Uncovering the mechanisms underlying SC quiescence provides opportunities for innovative therapeutic strategies. Such interventions fall into two main categories: “lock-out” strategies, which consist of pushing the cells out of the quiescent state to induce proliferation and differentiation; and “lock-in” strategies, which consist of re-establishing the quiescent state to prevent premature senescence or aberrant proliferation and differentiation (Figure 4). It has been argued that tumors are maintained by quiescent cancer stem cells (CSCs) and that, therefore, targeting quiescent cells could be a valuable approach to treating cancer (Chen et al., 2016).

#### “Lock-Out” Strategies

Several attempts have been made to genetically and pharmacologically induce activation of quiescent NSCs for tissue repair using a “lock-out” approach. For instance, depletion of neuroblasts and TAPs with the chemotherapeutic drug cytarabine stimulates rapid regeneration of the SVZ network (Doetsch et al., 1999), thought to be due to activation of the quiescent NSCs reservoir (Wang et al., 2011). A crucial role has also been reported for the Sonic hedgehog (Shh) pathway in switching quiescent NSCs to active NSCs within the SVZ. Shh induces an increase in the quiescent NSC pool as well as in the active NSC pool, followed by the complete depletion of active NSCs after long-term activation. These results highlight the vital role that Shh signaling plays in regulating NSC quiescence, which could be exploited to induce short-term neurogenesis to repair tissue damage.

Quiescence “lock-out” strategies have been employed in other tissues such as the skeletal muscle, where regenerative potential depends on MuSCs (Brack and Rando, 2012, Dumont et al., 2015). p38 MAPK signaling regulates the quiescent state of these cells, and upregulated signaling is observed in aged MuSCs. Inhibition of this signaling pathway leads to an increase in the number of quiescent MuSCs, improved self-renewal capacity, and better engraftment of aged MuSCs (Bernet et al., 2014).

Various novel cancer therapies also exploit quiescence “lock-out” strategies. For example, in patients with chronic myeloid leukemia, CSCs resistant to imatinib show high expression of *FBXW7*, a protein that promotes the degradation of c-Myc and Notch (Welcker and Clurman, 2008). The ablation of *FBXW7* in these cells dramatically enhances the effect of imatinib, inducing exit from the quiescent state and cell proliferation (Takeishi et al., 2013). Furthermore, *in vivo* administration of granulocyte colony-stimulating factor has been shown to induce human acute myeloid leukemia SCs to exit the quiescent state, enhancing their chemotherapeutic sensitivity (Saito et al., 2010). A recent study found that inhibition of macroautophagy by knockdown of autophagy-related 5 protein not only impairs the self-renewal capability of ovarian CSCs but also prevents them from entering quiescence and forces quiescent ovarian CSCs out of G_0_ (Wang et al., 2018).

#### “Lock-In” Strategies

“Lock-out” approaches for cancer treatment carry the risk of not eliminating all CSCs, leading to progression of the disease. To circumvent this, alternative “lock-in” strategies have been proposed to lock CSCs in G_0_, preventing tumor growth and metastasis. For instance, pharmacological inhibition of Src family kinase signaling combined with a MEK1/2 inhibitor prevents activation of metastatic quiescent breast CSCs by preventing progression to the G_1_ phase and inducing apoptosis (Mackay et al., 2012). Another study showed that BMP7, secreted by bone marrow stromal cells, causes cell-cycle arrest of prostate CSCs and confinement in a reversible quiescent state (Kobayashi et al., 2011).

 Approaches to re-establish SC quiescence have also been employed in muscle. Geriatric MuSCs undergo a controlled switch from quiescence to senescence, resulting in sarcopenia (loss of muscle mass and function) (Garcia-Prat et al., 2016). MuSC quiescence is retained through constitutive basal macroautophagy, but this mechanism deteriorates with age. By targeting the macroautophagy pathway through pharmacological intervention (with rapamycin or Trolox [6-hydroxy-2,5,7,8-tetramethylchroman-2-carboxylic acid]) or through overexpression of *Atg7*, quiescence can be re-established, reducing senescence and restoring the regenerative potential of MuSCs (Garcia-Prat et al., 2016). Maintenance of quiescence in MuSCs has also been attributed to repressed FGF2 signals. With age, FGF2 levels in skeletal muscle increase, inducing escape from quiescence and depletion of the MuSC pool (Chakkalakal et al., 2012). In cooperation with FGF2, β1-integrins are crucial in sustaining MuSC homeostasis, expansion, and self-renewal. Enhancing β1-integrin activity with a monoclonal antibody restored FGF2 sensitivity in aged MuSCs, leading to improved regeneration after muscle injury and in a model of Duchenne muscular dystrophy (Rozo et al., 2016).

An improved understanding of stem cell quiescence not only provides novel potential therapeutic interventions but could also improve previously established approaches. For instance, a key hurdle in autologous SC transplantation is the ability to manipulate the cells *in vitro* without a loss in potency. The potency of MuSCs correlates with their ability to remain quiescent, which is rapidly lost after isolation (Quarta et al., 2016). While some studies have attempted to recreate the biophysical environment of the MuSC niche, Quarta et al. (2016) identified a quiescent molecular signature and used it to develop a growth-free quiescence medium to culture isolated MuSCs. They also engineered artificial muscle fibers with optimal stiffness and elasticity to recapitulate that of native myofibers. These combined “lock-in” strategies maintained MuSC quiescence, which upon transplantation showed better engraftment, proliferation, self-renewal, and tissue repair (Quarta et al., 2016).

### Concluding Remarks

Quiescence is a state of reversible arrest in G_0_ that is actively maintained by various signaling inputs. Targeting quiescent stem cell populations is an attractive therapeutic strategy within the fields of oncology and regenerative medicine.

## Author Contributions

I.J.C., P.P.L., J.O., F.R., W.S., T.L.W., F.S., and F.M.W. conceived the manuscript. I.J.C., P.P.L., J.O., F.R., W.S., and T.L.W. wrote the manuscript. P.P.L., T.L.W., W.S., and F.R. designed the figures. J.O., T.L.W., and F.M.W. edited the manuscript.
